# Influence of airway pressure release ventilation on pulmonary gas exchange using ventilatory ratio

**DOI:** 10.3389/fmed.2026.1787967

**Published:** 2026-03-04

**Authors:** Emre Kiratlar, Patrick Rehn, Benjamin Neetz, Lars Reinhardt, Markus Alexander Weigand, Christoph Lichtenstern, Werner Schmidt, Armin Kalenka, Mascha O. Fiedler-Kalenka

**Affiliations:** 1 Department of Anesthesiology, Medical Faculty, University Heidelberg, Heidelberg University Hospital, Heidelberg, Germany; 2 Department of Pneumology and Critical Care Medicine, Thoraxklinik, Translational Research Center Heidelberg (TLRC-H), Member of the German Center for Lung Research (DZL), University of Heidelberg, Heidelberg, Germany; 3 German Center for Lung Research (DZL), Translational Lung Research Center Heidelberg (TLRC), Heidelberg, Germany; 4 Department of Anesthesiology and Intensive Care Medicine, Thoraxklinik, Heidelberg University Hospital, Heidelberg, Germany; 5 District Hospital Bergstrasse, Heppenheim, Germany

**Keywords:** acute respiratory distress syndrome, COVID-19, mechanical ventilation, time-controlled adaptive ventilation, ventilatory ratio

## Abstract

**Background:**

The COVID-19 pandemic led to a surge in Acute Respiratory Distress Syndrome (ARDS) cases. Despite important advances in ventilation strategies, ARDS mortality remains high. Airway Pressure Release Ventilation (APRV), especially when used according to the Time-Controlled Adaptive Ventilation (TCAV) protocol, has shown potential in improving oxygenation and reducing mortality in ARDS.

**Methods:**

This retrospective dual-center study included patients with moderate to severe ARDS, who were treated with APRV or Low Tidal Volume Ventilation (LTVV) between January 2018 and March 2022. Individuals receiving APRV for at least 72 h after previously receiving LTVV were analyzed in further detail. PaO_2_/FiO_2_ ratio and Ventilatory Ratio (VR) were measured 6, 12, 24, 48, and 72 h after transition to APRV. Statistical analyses were performed using univariate repeated measures ANOVA and chi-squared test.

**Results:**

Out of 107 patients, 48 received APRV. In 27 cases, APRV was applied according to TCAV-protocol. APRV was often used late in treatment or as a rescue therapy. Regarding the primary ventilation strategy, there was no significant difference in survival between APRV (44%) and LTVV (42%). In patients receiving APRV for at least 72 h after being initially ventilated with LTVV (*n* = 8), mean PaO_2_/FiO_2_ ratio improved significantly over time (*p* = 0.039), while mean VR decreased (*p* < 0.001).

**Conclusion:**

APRV demonstrated potential in improving gas exchange and ventilation efficiency in ARDS patients, particularly when used early and according to TCAV. However, no survival benefit was observed. The study’s retrospective design and heterogeneity in APRV application limit its conclusions.

## Background

The Coronavirus Disease 2019 (COVID-19) pandemic severely impacted patient care in Intensive Care Units (ICU) over the world as the disease can develop into an Acute Respiratory Distress Syndrome (ARDS) (1, 2). ARDS causes pulmonary edema, severely impaired gas exchange, and reduced lung compliance (3–5). As a result, invasive mechanical ventilation is often necessary to maintain sufficient gas exchange, which can in turn lead to aggravation of lung injury through excessive transpulmonary pressure or high tidal volumes resulting in Ventilator Induced Lung Injury (VILI) (6, 7). Despite current lung-protective ventilation strategies aiming to reduce VILI, mortality in ARDS remains high (8, 9).

“Time-Controlled Adaptive Ventilation” (TCAV) is a relatively new approach that utilizes Airway Pressure Release Ventilation (APRV) mode. TCAV can be conceptualized as continuous positive airway pressure (CPAP), that is intermittently interrupted by a release phase during which gas is expelled. The CPAP phase, which constitutes approximately 90% of the total respiratory cycle, allows for time-dependent lung recruitment. The release phase terminates when 75% of the expiratory peak flow is achieved (10, 11). Experimental studies have demonstrated that this approach restores lung volume while preventing VILI (12, 13). To date, there is a lack of studies that have tested the efficacy of TCAV in relation to patient-centered endpoints in a clinical setting (14).

Due to inflammation-induced microthrombi leading to microcirculatory disturbances and disruptions in hypoxic pulmonary vasoconstriction, ARDS can result in an increase in physiological dead space volume (15, 16). Numerous studies have identified increased dead space ventilation as an independent predictor of higher mortality in ARDS (15, 17, 18). The Ventilatory Ratio (VR) is an easy to obtain bedside index of ventilatory impairment. It exhibits a near-exponential relationship with physiological dead space fraction in subjects with ARDS and is associated with outcome (19, 20). VR is calculated by dividing the product of measured minute ventilation and measured PaCO₂ by the product of ideal minute ventilation and ideal PaCO₂ for the patient (19, 21, 22).


$$\text{Ventilatory Ratio}=\frac{\begin{array}{l} \text{Measured Minute}\;\\ \text{Ventilation}\;[\mathrm{ml}/\min]\;\\ \times \text{PaCO}₂[\text{mmHg}] \end{array}}{\begin{array}{l} \;\text{Predicted Body}\;\\ \text{Weight}\;[\mathrm{kg}]\;\\ \times 100\times 37.5\;\;\; \end{array}}≙\frac{\begin{array}{l} \text{Measured}\;\\ \text{Ventilation} \end{array}}{\begin{array}{l} \;\text{Ideal}\;\\ \text{Ventilation} \end{array}}$$

Under physiological conditions, VR is approximately 1. If pulmonary gas exchange is impaired, as in ARDS, a given minute ventilation may no longer be sufficient to achieve physiological PaCO_2_ values. Therefore, elevated VR values indicate ineffective ventilation with an inability to achieve adequate decarboxylation at given minute ventilation levels.

It was hypothesized that ventilation with APRV mode could achieve continuous, progressive lung tissue recruitment in ARDS patients over hours to days, while preventing subsequent de-recruitment, leading to corresponding reductions in VR and increases in P/F ratio over time. The aim of this dual-center retrospective cohort study was therefore to evaluate the effects of APRV on pulmonary gas exchange utilizing the VR to develop hypotheses for improving patient care and reducing morbidity.

## Methods

### Study population and data collection

**Figure 1:**
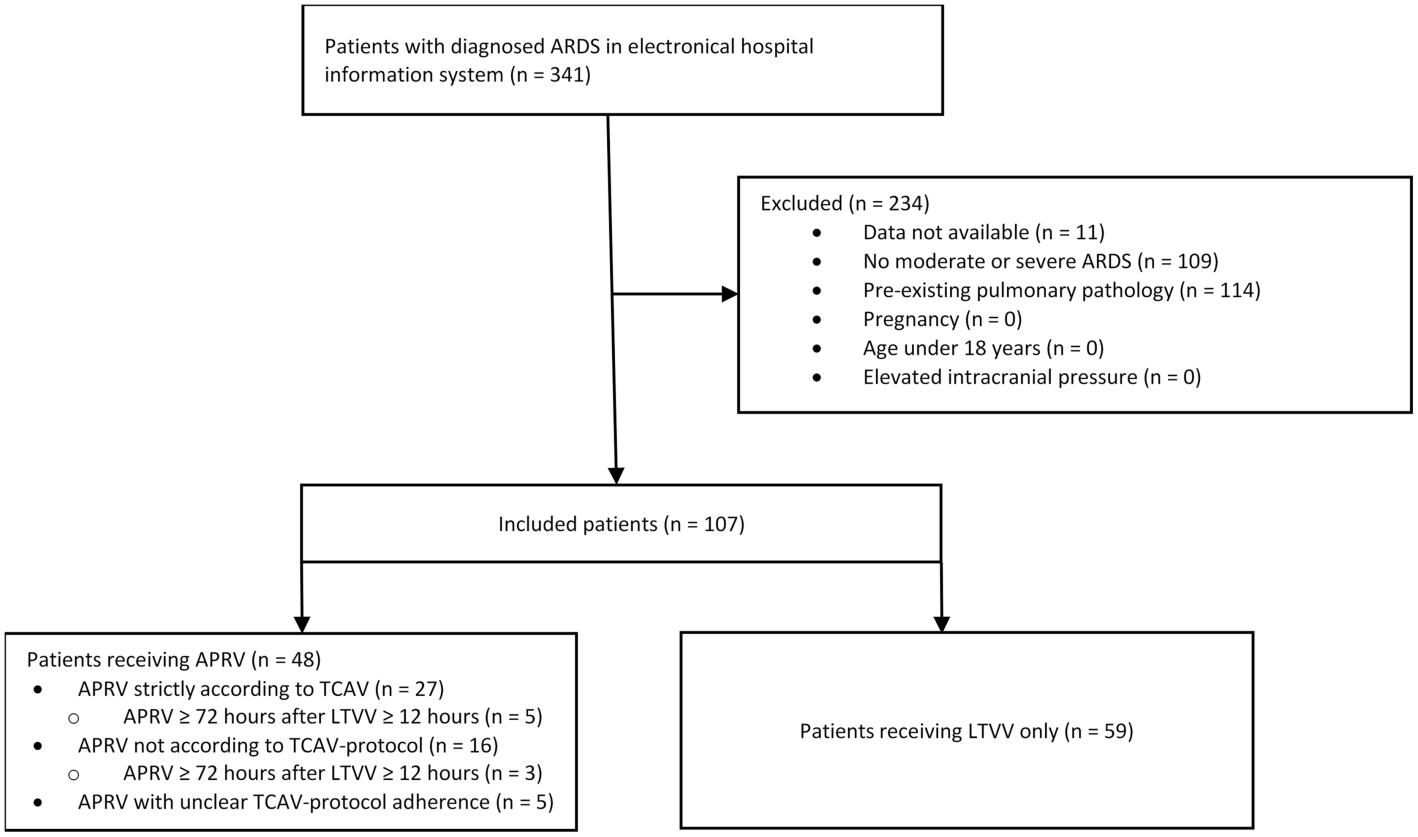
Inclusion and distribution of patients, consort diagram. ARDS, Acute Respiratory Distress Syndrome; TCAV, Time-Controlled Adaptive Ventilation; LTVV, Low Tidal Volume Ventilation.

All patients diagnosed with ARDS who received invasive mechanical ventilation for at least 24 h at the Interdisciplinary Surgical ICU of University Hospital Heidelberg and the ICU of Thoraxklinik of the University Hospital Heidelberg between January 2018 and March 2022 were included. The overall cohort consisted of 341 patients. Patients with mild ARDS were excluded (*n* = 109). For 11 patients, no sufficient data were available. All patients with pre-existing pulmonary pathology, including obstructive ventilatory disease, lung cancer, previous lung surgery or pulmonary fibrosis, were excluded from the study (*n* = 114). Other exclusion criteria were pregnancy (*n* = 0), age under 18 years (*n* = 0) and elevated intracranial pressure (*n* = 0). The analyzed cohort consisted of 107 patients (Figure 1).

All patient data were collected retrospectively by manual data extraction from electronic medical records (i.s.h.med, SAP® and COPRA PDMS, Copra System GmbH®). The extraction included epidemiological data, ventilation modes, comorbidities, and survival data. APRV characteristics were recorded in detail (P_high_, P_low_, T_high_, T_low_). Based on these data, it was evaluated whether or not the TCAV-protocol was used. If APRV was used early after ARDS-onset and served as the main ventilation strategy, it was classified as a “primary” use of APRV. However, if APRV was used as an alternative therapeutic attempt in the context of a pulmonary or overall critical clinical situation, it was labeled as a “rescue attempt.” Patients who were ventilated with Low Tidal Volume Ventilation (LTVV = ventilation with tidal volumes of 6 mL/kg of predicted body weight) for at least 12 h and were then transitioned to APRV for at least 72 continuous hours were examined more closely. P/F-ratio (PaO_2_/FiO_2_) and VR were analyzed 6, 12, 24, 48, and 72 h after transitioning to APRV. Baseline P/F-ratio and VR were defined as the average values from the 12 h preceding the transition.

### Ethics statement

Ethical approval for this study (reference number S-298/2022) was provided by the local Ethics Committee of the Medical Faculty of Heidelberg University, Alte Glockengießerei 11/1, 69115 Heidelberg, Germany, on August 26th, 2022.

### Endpoints

The primary endpoints were overall in-hospital-mortality comparing LTVV and APRV and the changes in P/F ratio and VR at 6, 12, 24, 48, and 72 h after the transition to APRV in the patients included in this analysis. The secondary endpoints were the evaluation of APRV usage in general and the assessment of TCAV-protocol implementation.

### Statistical analysis

Statistical analyses were conducted using the software IBM SPSS Statistics (version 29.0; International Business Machines Corporation).

Normal distributions of the variables were assessed using histograms with normal distribution curves, Q-Q plots, and Shapiro–Wilk tests. Outliers were evaluated for their content validity and, if deemed plausible, included in the analyses. Univariate repeated measures ANOVA was used to analyze P/F-ratio and VR after transitioning to APRV, chi-squared test was used for overall mortality. Because of a violation of normal distribution due to a right-skewed distribution of the values, the absolute values of the P/F-ratios were logarithmically transformed. To check for sphericity, the Mauchly test was applied. After the analysis of the main effect, Bonferroni-corrected pairwise comparisons between the individual time points were conducted as post-hoc tests. The level of significance was set at 0.05.

## Results

A total of 107 patients were included in the study. In 48 patients, APRV was used at least once, whereas in 59 patients, only LTVV was used. Overall patient characteristics are presented in Table 1.

**Table 1:** Overall patient characteristics.

Characteristic	All patients	APRV used at least once	Only LTVV
Total patients	107	48	59
Male [*n*]	73 (68%)	34 (71%)	39 (66%)
COVID-19 positive [*n*]	59 (55%)	42 (88%)	17 (29%)
VV-ECMO [*n*]	34 (32%)	23 (48%)	11 (19%)
ASA 1 [*n*]	4 (4%)	3 (6%)	1 (2%)
ASA 2 [*n*]	36 (34%)	19 (40%)	17 (29%)
ASA 3 [*n*]	42 (39%)	23 (48%)	19 (32%)
ASA 4 [*n*]	24 (22%)	4 (8%)	21 (36%)
ASA 5 [*n*]	1 (1%)	0	1 (2%)
Survived hospital stay [*n*]	40 (37%)	16 (33%)	24 (41%)
Death within hospital stay [*n*]	54 (51%)	30 (63%)	24 (41%)
Lost to follow-up [*n*]	13 (12%)	2 (4%)	11 (19%)
Age [years]	63.0 (15.2/107)	61.4 (14.3/48)	64.4 (15.8/59)
BMI [kg/m^3^]	30.1 (8.9/97)	32.2 (9.5/46)	28.2 (8.0/51)
SAPS on admission	48.8 (19.4/107)	45.8 (17.6/48)	51.2 (20.5/59)
Length of ICU stay [days]	38.5 (34.5/91)	48.6 (42.4/43)	29.5 (22.3/48)
Length of mechanical ventilation [days]	26.0 (24.0/96)	34.2 (27.8/44)	19.1 (17.7/52)

For metric variables, the arithmetic means are provided. The first number in the parentheses indicates standard deviation, the second number shows for how many patients data were available for this variable.

LTVV, Low Tidal Volume Ventilation; APRV, Airway Pressure Release Ventilation; COVID-19, Corona Virus Disease 2019; VV-ECMO, Veno-Venous Extracorporeal Membrane Oxygenation; ASA, American Society of Anesthesiologists; ICU, Intensive Care Unit; BMI, Body Mass Index; SAPS, Simplified Acute Physiology Score.

### Usage of APRV

**Figure 2:**
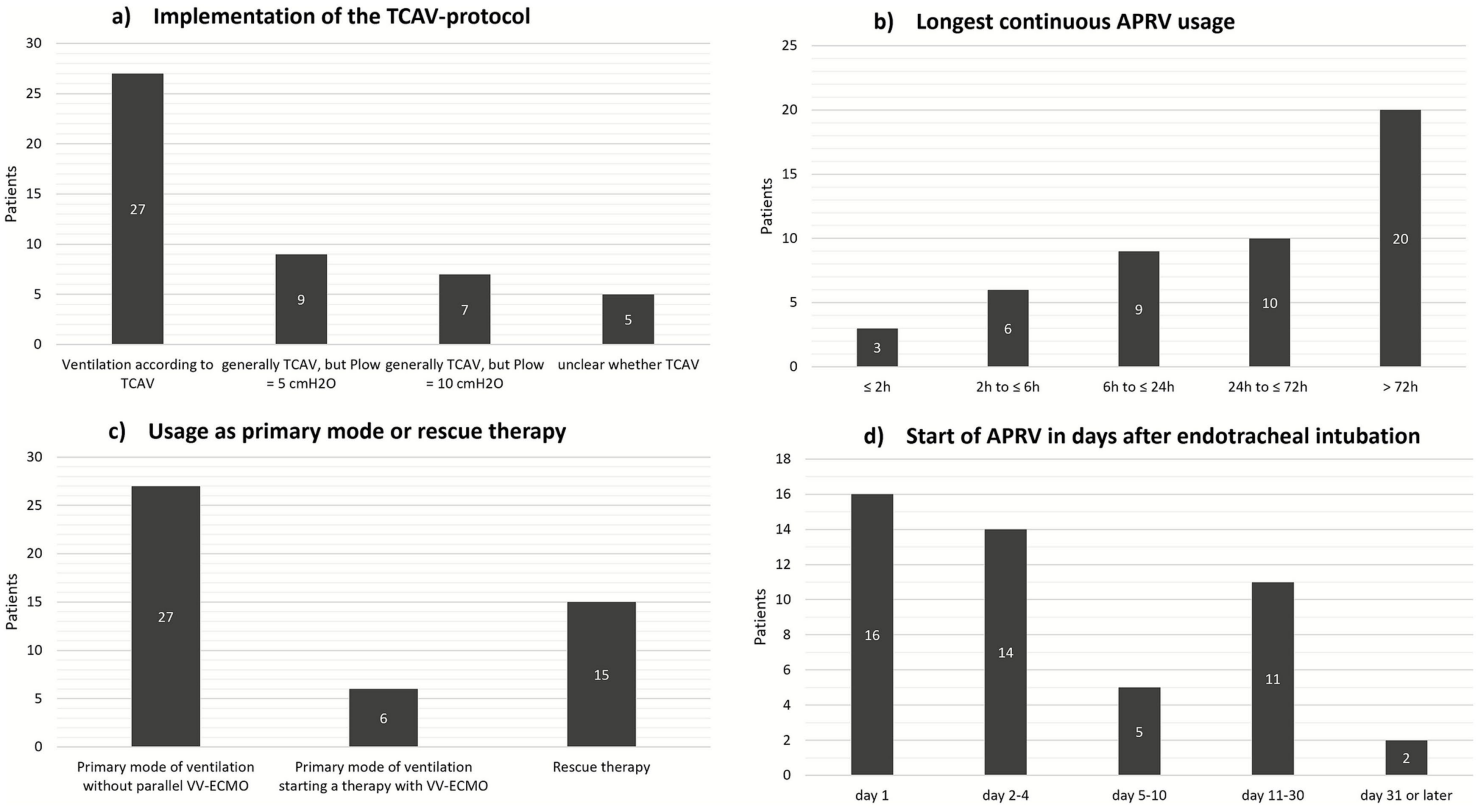
Details of APRV usage. TCAV, Time-Controlled Adaptive Ventilation; APRV, Airway Pressure Release Ventilation; VV-ECMO, Veno-venous extracorporeal membrane oxygenation. **(a)** Implementation of TCAV protocol, **(b)** Longest continuous APRV usage, **(c)** Usage of APRV as primary mode or rescue therapy, **(d)** Start of APRV after endotracheal intubation.

Overall, 48 patients were ventilated with APRV. Among these patients, 27 were ventilated strictly according to TCAV protocol. TCAV protocol deviation was observed in 16 patients ventilated with APRV (P_low_ was set to 5 or 10 cmH_2_O instead of 0 cmH_2_O). In 5 cases, adherence to the TCAV-protocol could not be verified due to imprecise documentation of ventilation parameters (Figure 2a). Twenty patients received APRV for at least 72 consecutive hours, whereas in 9 cases, APRV was discontinued after a maximum of 6 h, mainly due to a lack of improvement in the clinical situation during a rescue attempt (Figure 2b). APRV was used as the primary mode of ventilation in 27 patients. In 15 patients, APRV was used as a rescue strategy (Figure 2c). In 13 patients, APRV was applied on the eleventh day of mechanical ventilation or later (Figure 2d).

### Overall in-hospital mortality

Excluding all patients lost to follow-up, 24 out of 48 patients (50%) survived when only LTVV was applied, whereas 16 out of 46 patients (34.8%) survived, when APRV was used at any point (Table 2). There was no significant difference in overall survival between these two groups, χ^2^(1) = 2.23, *p* = 0.136, *φ* = 0.15. Excluding all patients lost to follow-up, 29 out of 69 patients (42%) survived when LTVV was the primary ventilation strategy, whereas 11 out of 25 patients (44%) survived when APRV was the primary ventilation strategy (Table 2). There was no significant difference between the two groups, χ^2^(1) = 0.29, *p* = 0.864, φ = 0.018.

**Table 2:** Overall in-hospital mortality.

Outcome	LTVV only	APRV at least once	LTVV as primary ventilation mode^a^	APRV as primary ventilation mode^a^
Survived	24	16	29	11
Deceased	24	30	40	14
Lost to follow-up	11	2	11	2

a If APRV was not used as the primary ventilation strategy but rather as a rescue therapy or was first used under VV-ECMO, the patient was assigned to the “LTVV as primary ventilation mode” group. If APRV was used as the main ventilation strategy, the patient was assigned to the “APRV as primary ventilation mode” group.

LTVV, Low Tidal Volume Ventilation; APRV, Airway Pressure Release Ventilation.

### Pulmonary gas exchange after transition to APRV

Only eight patients could be included in this analysis, for whom complete data for the specified period were available. Five of these patients were ventilated strictly according to TCAV-protocol, whereas in 3 patients, TCAV-settings for P_high_, T_low_, and T_high_ were applied, but P_low_ was set at 5 cmH₂O. None of the patients required venovenous extracorporeal membrane oxygenation (VV-ECMO) during the 12-h LTVV or the consecutive 72-h APRV observation period. During the further course of treatment (after observation period), 4 patients received VV-ECMO therapy. Diagnoses included COVID-19 pneumonia (*n* = 7) and miliary tuberculosis (*n* = 1). Individual patient data, P/F-ratio and VR over the course of the observation period is reported in the supplements (Supplementary Tables S1, S2; Supplementary Figures S1, S2).

### P/F ratio

**Figure 3:**
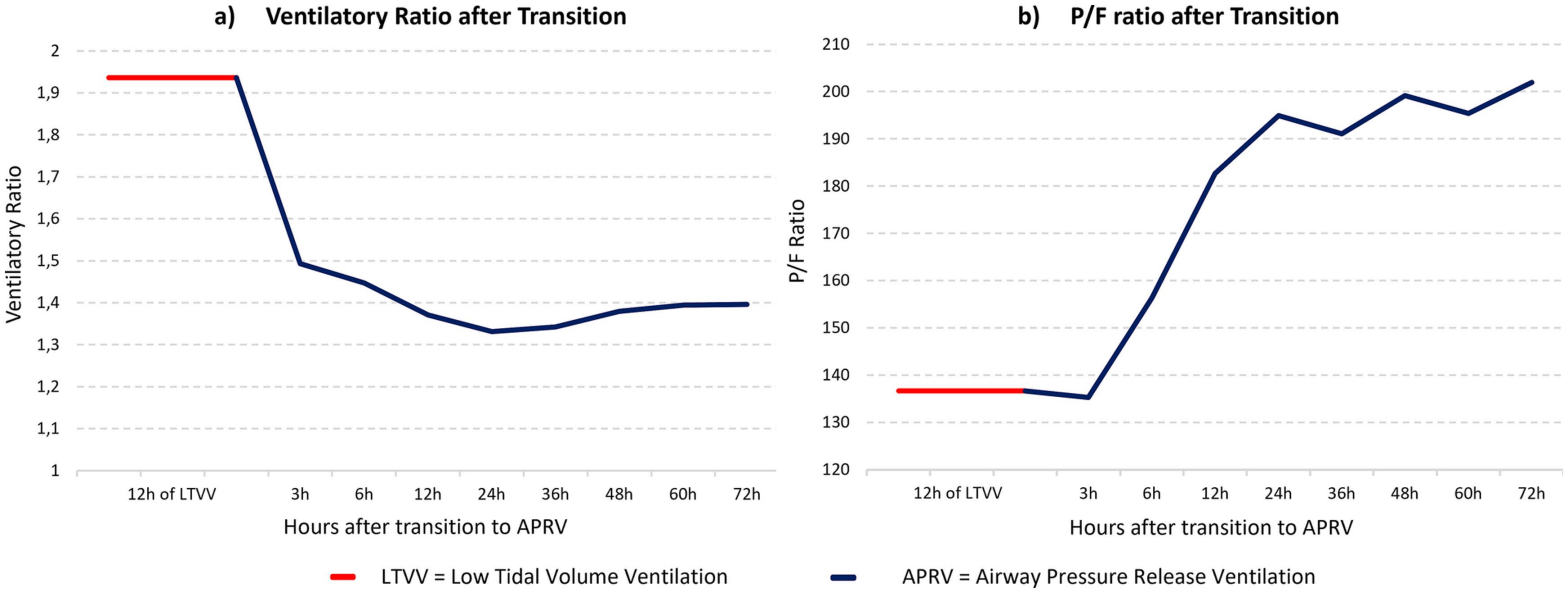
Gas exchange parameters after transition to APRV (*n* = 8). APRV, Airway Pressure Release Ventilation; LTVV, Low Tidal Volume Ventilation; P/F ratio, pO2/FiO2 ratio. **(a)** Ventilatory Ratio after Transition to APRV, **(b)** P/F ratio after Transition to APRV.

The mean P/F ratio was lowest before the transition to APRV and increased continuously in the following hours, as shown in Table 3 and Figure 3b.

**Table 3:** Mean P/F ratio after transition to APRV.

Parameter	Prior to transition	6 h	12 h	24 h	48 h	72 h
Mean P/F ratio [mmHg]	136.7 (SD = 42.6)	156.3 (SD = 39.7)	182.7 (SD = 59.0)	194.9 (SD = 52.3)	199.2 (SD = 78.1)	201.9 (SD = 71.1)
Mean ln (P/F ratio)	4.88 (SD = 0.30)	5.03 (SD = 0.24)	5.17 (SD = 0.30)	5.24 (SD = 0.28)	5.22 (SD = 0.44)	5.25 (SD = 0.38)

Time points are expressed as hours after transition to APRV; SD, Standard Deviation; APRV, Airway Pressure Release Ventilation; P/F ratio, pO2/FiO2 ratio.

A repeated measures ANOVA revealed that the mean P/F ratio differed significantly over time, *F*(5, 35) = 2.65, *p* = 0.039, partial η^2^ = 0.28. However, Bonferroni-corrected pairwise comparisons between the individual time points revealed no significant differences (*p* > 0.05).

### Ventilatory ratio

Mean VR was highest before the transition and reached its lowest value 24 h after the transition, as shown in Table 4 and Figure 3a.

**Table 4:** Mean ventilatory ratio after transition to APRV.

Parameter	Prior to transition	6 h	12 h	24 h	48 h	72 h
Mean ventilatory ratio	1.94 (SD = 0.32)	1.45 (SD = 0.27)	1.37 (SD = 0.19)	1.33 (SD = 0.22)	1.38 (SD = 0.28)	1.40 (SD = 0.38)

Time points are expressed as hours after transition to APRV; SD, Standard Deviation; APRV, Airway Pressure Release Ventilation.

The assumption of sphericity was violated according to the Mauchly test (*p* = 0.004). Therefore, a Greenhouse–Geisser correction was applied. A corresponding repeated measures ANOVA revealed that the mean VR differed significantly over time, *F*(2.08, 14.56) = 17.80, *p* < 0.001, partial η^2^ = 0.72.

Bonferroni-corrected pairwise comparisons between the individual time points revealed a significant difference between the mean VR before the transition and the mean VR at any time point after the transition. The corresponding *p*-values are presented in Table 5. No significant differences were found in any pairwise comparisons between the individual time points after the transition to APRV (*p* > 0.05).

**Table 5:** *p*-values of the pairwise comparisons between mean VR before transition and mean VR at specific time points after transition.

Reference	6 h	12 h	24 h	48 h	72 h
Prior to transition	*p* < 0.001 (*M*_Diff_ = 0.49)	*p* < 0.001 (*M*_Diff_ = 0.57)	*p* = 0.002 (*M*_Diff_ = 0.61)	*p* = 0.003 (*M*_Diff_ = 0.56)	*p* = 0.04 (*M*_Diff_ = 0.54)

VR, Ventilatory Ratio; *M*_Diff_, Mean Difference.

## Discussion

In this retrospective cohort study of patients with moderate to severe ARDS, we found that overall in-hospital-mortality did not differ between ventilation with APRV and ventilation with LTVV. However, we found improved oxygenation and indications of reduced dead space ventilation and higher ventilation efficiency when patients were transitioned to APRV after prior ventilation with LTVV.

To capture the effects of APRV over an extended period, a duration of 72 h of continuous APRV following at least 12 h of observed LTVV was chosen. There was a statistically significant effect on the P/F ratio over time. After transition to APRV, mean P/F ratio at 24 h was 43% higher than before the transition.

Numerous studies have reported a positive effect of APRV on oxygenation (23–26). The observed increase is likely attributable to enhanced alveolar recruitment and stabilization. Gradual opening and stabilization of opened alveoli are key elements of TCAV. Repetitive alveolar collapse and expansion (RACE) is effectively prevented (27–29). Prolonged CPAP-Phases and terminating P_low_ at 75% of expiratory peak flow has shown to be the most effective way to achieve this stability when using APRV (30). Strict protocol adherence when ventilating patients using TCAV is therefore paramount. Although the average P/F ratio increased, this effect was not equally strong in every patient. Individual patient trajectories demonstrated a heterogeneous response. In 4 of 8 patients an increase in P/F-Ratio of ≥50 mmHg within 24 h could be seen, whereas the remaining patients showed minimal or no improvement. This responder distribution suggests that mean effects were driven by clinically meaningful improvements in a subset of patients rather than uniform small changes across the cohort. A common characteristic among patients who improved was that the transition to APRV occurred within the first 72 h following endotracheal intubation. In contrast, all 4 patients with little to no improvement in oxygenation were transitioned to APRV on the third day of mechanical ventilation or later. Particularly in this early phase of ARDS, inappropriate ventilation patterns and mechanical stress can damage the vulnerable lung (31). In these 4 patients, VILI may have already developed to some extent as a result of atelectrauma through RACE caused by LTVV. Due to viscoelastic properties of the lung, the short release phase used in TCAV can prevent de-recruitment of opened lung areas and achieve alveolar stabilization (32). Therefore, early application of TCAV has the potential to prevent the progression of ARDS and VILI, thereby facilitating enhanced lung healing (33, 34).

After transition to APRV, there was a significant reduction in VR. This reduction was already statistically significant after 6 h. The mean VR approached a value of 1.0 after the transition to APRV. This implies that, with a constant minute ventilation, more CO_2_ could be eliminated, or alternatively, a lower minute ventilation was sufficient to achieve a defined CO_2_ elimination. Consequently, a lower VR could be interpreted as an indicator of more efficient ventilation under APRV compared to LTVV. One potential reason for this effect could be improved lung tissue recruitment and prevention of de-recruitment, resulting in an increase in physiologically active alveolar surface area. An additional explanation is the diffusion of CO₂-containing respiratory gas from the alveolar compartment toward the central airways during the prolonged CPAP-phase (35). This effectively reduces the physiological dead space and achieves a VR that is otherwise only observed in patients with healthy lungs. Similar to the present study, some retrospective studies have investigated VR after initiation of APRV. Comparable to our results, these studies reported a decrease in mean PaCO_2_ despite a lower minute ventilation and, consequently, a reduction of the VR (36, 37). It should be noted that in studies demonstrating a correlation between physiological dead space ventilation and VR, mechanical ventilation was exclusively performed using LTVV (19, 22). Therefore, it remains questionable to what extent VR can also estimate dead space ventilation in APRV. Nevertheless, a VR closer to 1.0 suggests higher efficiency in the physiological utilization of the applied minute ventilation. In our study, this assumption is further supported by the fact, that VR continued to show a decreasing trend even after 6 h, reaching its lowest value after 24 h. Similar to the decreasing VR, the P/F ratio showed a steady increase in the hours following the transition. This dynamic development, with continuous improvement in both oxygenation and CO_2_ elimination under APRV, could imply ongoing recruitment of collapsed alveolar surface area. Our data indicate that maximum lung recruitment is achieved within a period of approximately 24 h. While strategies aimed at alveolar recruitment over seconds or minutes could not achieve significant success, a continuous recruitment over hours or even days might be the optimal method to reopen collapsed lung areas (38, 39). This gradual reopening of the de-recruited lung might be less damaging to the injured lung than previously published approaches (27).

Some studies have demonstrated lower mortality with APRV compared to LTVV (24–26). However, this association could not be reproduced in the present study. A potential explanation for this could be the retrospective study design, which lacked a standardized experimental protocol and randomization of the therapeutic strategies. This could have led to many difficult-to-measure covariates, which might have significantly influenced mortality. Furthermore, the sample size was small.

When APRV was used as a rescue therapy, only 2 out of 15 patients survived the remainder of their ICU stay. In these patients, pulmonary function had already been severely impaired, immensely worsening the clinical prognosis. In such cases, it is reasonable to conclude that APRV was unlikely to facilitate a significant recovery.

It has to be noted that there was no standard operating procedure regarding the use of APRV during the observed time period in the included ICUs. This resulted in APRV being used in heterogeneous situations and settings. Although TCAV was used as a template by the practitioners, in some cases, P_low_ was not set to 0 cmH_2_O. In TCAV, the end-expiratory lung volume is not determined by the extrinsically applied PEEP, but by the short expiratory time (15). A P_low_ set higher than 0 cmH_2_O can significantly affect the expiratory airflow and undermine the underlying principles of TCAV (40).

### Strengths and limitations

The primary strength of this study lies in its ability to minimize significant interindividual differences. By excluding patients with pre-existing pulmonary conditions, a homogenous patient cohort could be established. Furthermore, the analysis of multiple ventilation strategies in the same patients allowed for an examination of the individual effects of APRV.

There are various weaknesses and limitations regarding this study. The retrospective design significantly restricts the prognostic validity of the obtained data. Many relevant parameters and potential confounding factors could only be assessed with limited accuracy. APRV was not always applied strictly according to the TCAV-protocol. Additionally, APRV was sometimes discontinued prematurely, potentially resulting in a selective group of patients being included in the analyses.

Furthermore, APRV was not routinely applied to every patient. It was often used as a rescue strategy in patients with severe impairment of gas exchange leading to potential selection bias. Additionally, high COVID-19 prevalence in this cohort limits generalizability. A further limitation is the inability to categorize patients by ARDS phenotype. Due to missing data on physiological markers such as recruitability and compliance and biological markers such as inflammation-markers, no definitive conclusions can be drawn regarding which patient subsets might best respond to APRV.

The analysis of gas exchange following transition to APRV was limited to a small subset of patients (*n* = 8), which is insufficient to provide adequate statistical power for definitive conclusions in a heterogeneous ARDS population. Consequently, these findings should be interpreted as hypothesis-generating rather than confirmatory. No conclusions can be drawn for any patients with pre-existing pulmonary conditions.

## Conclusion

Our study highlights the potential of APRV to enhance gas exchange in patients with moderate to severe ARDS. Based on the observed changes in blood gasses over time, it can be concluded that APRV may potentially recruit alveolar surface area, reduce dead space ventilation, improve oxygenation, and enhance ventilation efficiency. With TCAV, there is an established, easy-to-use protocol which incorporates fundamental physiological advantages and has shown promising results in recent studies. Taken together, these findings suggest that APRV, particularly when applied early and according to TCAV principles, may improve oxygenation and ventilation efficiency in selected ARDS patients. However, given the small sample size, these results should be viewed as hypothesis-generating and warrant confirmation in adequately powered prospective trials.

## Data Availability

The raw data supporting the conclusions of this article will be made available by the authors, without undue reservation.

## Abbreviations

ANOVA: Analysis of variance
APRV: Airway pressure release ventilation
ARDS: Acute respiratory distress syndrome
ASA: American society of anesthesiologists
BMI: Body mass index
COVID-19: Corona virus disease 2019
FiO_2_: Fraction of inspired oxygen
ICU: Intensive care unit
LTVV: Low tidal volume ventilation
PaCO_2_: Arterial partial pressure of carbon dioxide
PaO_2_: Arterial partial pressure of oxygen
PBW: Predicted body weight
PEEP: Positive end-expiratory pressure
P/F ratio: Ratio of the arterial partial pressure of oxygen divided by the fraction of inspired oxygen (PaO_2_/FiO_2_)
P_high_: Higher pressure level in mechanical ventilation
P_low_: Lower pressure level in mechanical ventilation
SAPS: Simplified acute physiology score
TCAV: Time-controlled adaptive ventilation
T_high_: Time on higher pressure level in mechanical ventilation
T_low_: Time on lower pressure level in mechanical ventilation
VILI: Ventilator induced lung injury
VR: Ventilatory ratio
VV-ECMO: Veno-venous extracorporeal membrane oxygenation
