# Supplementary material for: Influence of airway pressure release ventilation on pulmonary gas exchange using ventilatory ratio
Source: Front Med (Lausanne). 2026 Mar 4;13:1787967. doi: 10.3389/fmed.2026.1787967 (PMC12996203; doi:10.3389/fmed.2026.1787967)
Supplement: Supplementary file 1 [file Supplementary_file_1.docx]

**Suppl. Table 1 | Overall characteristics for included patients in gas exchange analysis with transition to APRV**

|  |  |
| --- | --- |
| Total patients | 8 |
| Male | 5 |
| Female | 3 |
| COVID-19 pneumonia | 7 |
| Miliary tuberculosis | 1 |
| Active VV-ECMO during observation period | 0 |
| P_low_ = 0cmH_2_O (TCAV) | 5 |
| P_low_ = 5cmH_2_O | 3 |
| Age [years] | 49.5 (13.8) |
| BMI [kg/m³] | 33.5 (9.3) |
| Baseline P/F ratio | 136.7 (42.6) |
| P/F ratio at 72h APRV | 201.9 (71.1) |
| Baseline VR | 1.94 (0.32) |
| VR at 72h APRV | 1.40 (0.38) |

For metric variables, the arithmetic mean is provided. The number in the parentheses indicates standard deviation.

APRV = Airway Pressure Release Ventilation, COVID-19 = Corona Virus Disease 2019, VV-ECMO = Veno-Venous Extracorporeal Membrane Oxygenation, P_low_ = lower pressure level in mechanical ventilation, TCAV = Time-Controlled Adaptive Ventilation, BMI = Body Mass Index, VR = Ventilatory Ratio

**Suppl. Table 2 | Individual patient data** **for included patients in gas exchange analysis with transition to APRV**

| Patient | Age [years] | Sex | BMI [kg/m³] | COVID-19 | TCAV adherence | Days to APRV | Baseline P/F | P/F at 72h APRV | Baseline VR | VR at 72h APRV |
| --- | --- | --- | --- | --- | --- | --- | --- | --- | --- | --- |
| 1 | 56 | F | 43,5 | Yes | P_low_ = 0cmH_2_O | 2 | 79,4 | 240,0 | 1,60 | 1,64 |
| 2 | 55 | M | 35,1 | Yes | P_low_ = 5cmH_2_O | 2 | 130,8 | 217,8 | 2,44 | 1,91 |
| 3 | 49 | M | 27,2 | Yes | P_low_ = 0cmH_2_O | 3 | 128,4 | 152,9 | 1,79 | 1,13 |
| 4 | 23 | F | 27,3 | No | P_low_ = 0cmH_2_O | 3 | 125,5 | 197,7 | 1,70 | 0,89 |
| 5 | 72 | F | 50,9 | Yes | P_low_ = 5cmH_2_O | 3 | 108,5 | 262,1 | 2,36 | 1,71 |
| 6 | 46 | M | 24,2 | Yes | P_low_ = 0cmH_2_O | 4 | 151,9 | 109,2 | 1,83 | 0,93 |
| 7 | 43 | M | 28,9 | Yes | P_low_ = 5cmH_2_O | 4 | 226,7 | 314,2 | 2,10 | 1,39 |
| 8 | 52 | M | 30,9 | No | P_low_ = 0cmH_2_O | 7 | 142,3 | 121,5 | 1,67 | 1,57 |

APRV = Airway Pressure Release Ventilation, F = female, M = male, COVID-19 = Corona Virus Disease 2019, BMI = Body Mass Index, TCAV = Time-Controlled Adaptive Ventilation, P_low_ = lower pressure level in mechanical ventilation, VR = Ventilatory Ratio


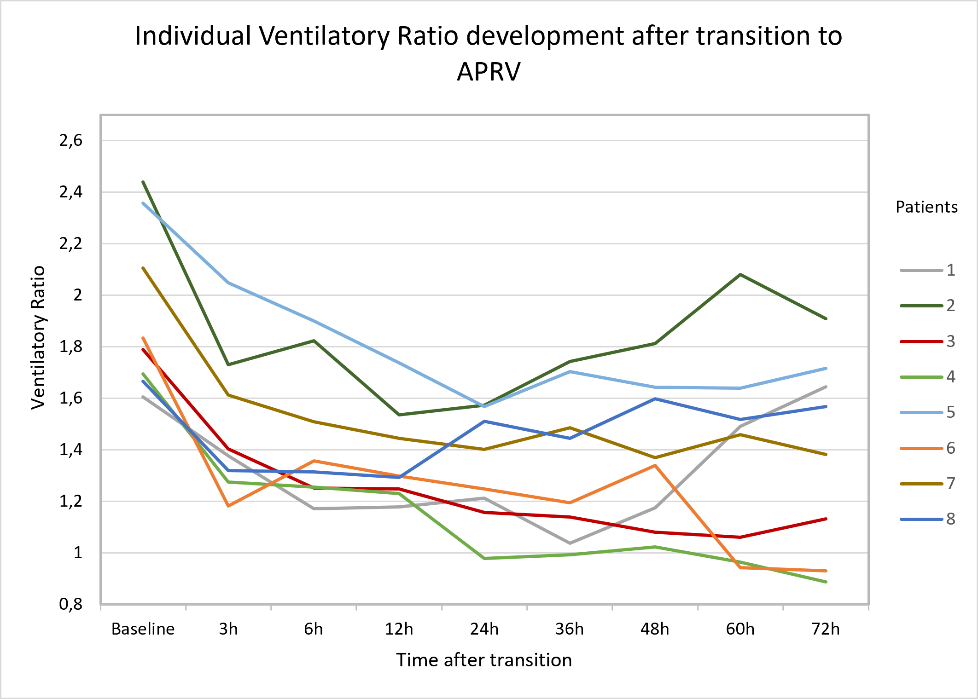


Suppl. Figure 1: Ventilatory Ratio (VR) for each patient included in gas exchange analysis with transition to APRV. Each color represents one individual patient.


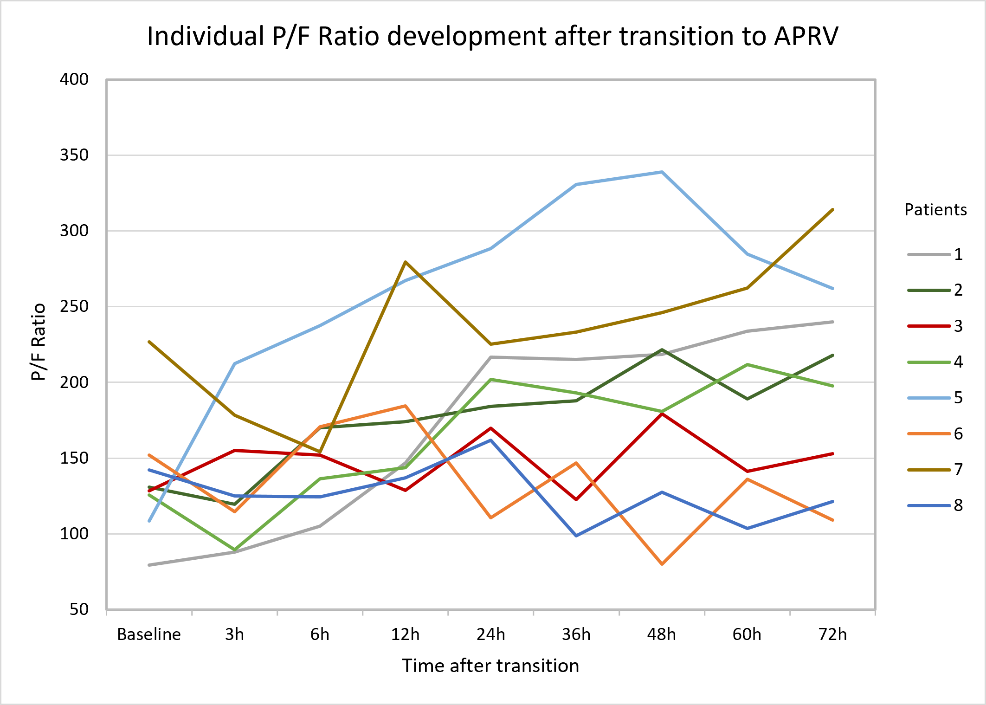


Suppl. Figure 2: PO2-FiO2-Ratio (P/F-Ratio) for each patient included in gas exchange analysis with transition to APRV. Each color represents one individual patient.
